# Effect of Long-Term Outdoor Storage on the Multi-Scale Structural and Mechanical Properties of Oriental Beech (*Fagus orientalis* Lipsky) and European Hornbeam (*Carpinus betulus* L.) Wood

**DOI:** 10.3390/polym18151915

**Published:** 2026-08-05

**Authors:** Göksu Şirin

**Affiliations:** Department of Forestry and Forest Products, Almus Vocational School, Tokat Gaziosmanpaşa University, Tokat 60900, Türkiye; goksu.sirin@gop.edu.tr

**Keywords:** *Carpinus betulus*, *Fagus orientalis*, mechanical properties, outdoor storage, thermal stability, wood degradation, X-ray diffraction

## Abstract

This study examines the effects of nearly six years of outdoor storage on the chemical, thermal, structural, micromorphological, and mechanical properties of European hornbeam (*Carpinus betulus* L.) and Oriental beech (*Fagus orientalis* Lipsky) wood. Differences between fresh (control) and stored samples were characterised using attenuated total reflectance–Fourier transform infrared (ATR-FTIR) spectroscopy, thermogravimetric/derivative thermogravimetric analysis (TGA/DTG), X-ray diffraction (XRD), scanning electron microscopy (SEM), and testing of compressive strength parallel to the grain. ATR-FTIR analysis points to alterations in carbohydrate- and lignin-related absorption bands after storage. TGA/DTG profiles suggest that hornbeam largely maintains its thermal degradation behaviour, whereas beech exhibits more pronounced shifts in thermal stability. XRD results indicate a reduction in crystallinity index (CrI) for both species. SEM observations of beech wood revealed filamentous structures within cell lumens, together with localised cell-wall disruption and microcracks, indicating more extensive microstructural modification than in hornbeam. Mechanical testing shows significant reductions in modulus of elasticity (MOE), with decreases of 32.2% in hornbeam and 28.4% in beech. Statistical evaluation confirms the significance and effect sizes of these changes. Overall, the findings demonstrate that long-term outdoor storage induces species-dependent degradation across molecular, microstructural, and macroscopic scales, ultimately compromising wood performance.

## 1. Introduction

Wood, as one of the primary representatives of biomass, is an abundant and renewable lignocellulosic engineering material [1]. The physical, chemical, and mechanical properties of wood are governed by the proportions of its main structural components within the polymer matrix (cellulose, hemicelluloses, lignin, and low-molecular-weight extractives) as well as their morphological organisation and mutual interactions [2]. Owing to this complex structure, wood has long been utilised as a strategic raw material across various industrial sectors.

In the forest industry, the outdoor storage of logs for extended periods is a common and often inevitable practice to secure supply continuity, balance seasonal harvest fluctuations, and manage climate-driven supply uncertainties [3,4]. However, this storage process exposes the material to environmental factors, often triggering a progressive degradation cycle over time. Abiotic factors such as fluctuations in temperature and relative humidity, UV radiation, and oxidation alongside biotic agents like fungal and insect attacks [5,6], lead to dry matter losses in the stored raw material, the weakening of cell wall integrity, and ultimately, a decline in mechanical performance [7,8,9,10]. The literature indicates that the severity of this structural degradation under outdoor conditions is directly correlated with climatic variables, storage duration, and the anatomical and chemical profile of the wood species [6,11,12]. Particularly during long-term exposure, it has been reported that the breakdown of the carbohydrate matrix (primarily hemicelluloses) accelerates, cellulose crystallinity decreases, and macroscopic cracks develop on the material surface [3,13].

While controlled laboratory experiments provide valuable theoretical insights into degradation mechanisms, field-based observations that represent real service conditions offer a much more reliable perspective for evaluating the effects of long-term storage [14,15,16]. Therefore, elucidating the behaviour of different wood species under natural storage conditions provides essential input for developing appropriate inventory management strategies, optimising storage durations, and minimising economic losses [17,18].

European hornbeam (*Carpinus betulus* L.) and Oriental beech (*Fagus orientalis* Lipsky), which are widely distributed across the ecosystems of the Black Sea, the Caucasus, and Europe, are among the most significant hardwood species providing high value-added products in the regional forest products industry [19,20]. European hornbeam is characterised by its high density, hardness, and mechanical strength [21,22], whereas Oriental beech is noted for its good workability and elastic properties [9,23]. Despite these specific differences in their anatomical and physicomechanical properties, both species are often utilised in similar industrial applications. Table 1 summarises the key physical and chemical characteristics of these species reported in the literature.

The aim of this study was to characterise the chemical, thermal, crystalline, microstructural, and mechanical changes occurring in Oriental beech and European hornbeam wood following long-term exposure to outdoor conditions in forest storage yards without preservative treatment, by integrating complementary analyses across multiple structural scales. The wood specimens evaluated in this study were obtained from materials naturally stored under outdoor environmental conditions for nearly six years. Although the properties of fresh wood of both species have been extensively investigated, studies evaluating the effects of long-term natural storage on their structural integrity and performance remain rather limited.

Accordingly, storage-induced changes in the functional groups of lignocellulosic components were examined by attenuated total reflectance–Fourier transform infrared spectroscopy (ATR-FTIR), changes in thermal stability by thermogravimetric analysis/derivative thermogravimetry (TGA/DTG), variations in the crystalline structure of cellulose by X-ray diffraction (XRD), and cellular-level morphological deformations by scanning electron microscopy (SEM). The resulting chemical, thermal, crystalline and microstructural findings were comparatively evaluated together with the compressive strength parallel to the grain and modulus of elasticity (MOE), representing the macroscopic performance of the material.

## 2. Materials and Methods

### 2.1. Collection and Preparation of Wood Specimens

In this study, two experimental groups were established for European hornbeam and Oriental beech. The first group (Stored) comprised logs stored in a forest log yard located in Tokat province, Türkiye (40°20′34.93″ N, 36°53′44.64″ E; altitude: 850.6 m). The study area is characterised by a continental climate with hot, dry summers and cold, rainy winters accompanied by moderate to strong winds. The region receives an average annual precipitation of 392 mm, with annual temperatures ranging from −25 °C to 41 °C [33]. The European hornbeam and Oriental beech logs were placed in the yard on 28 January 2019 and 18 January 2019, respectively. Both species were retrieved on 2 December 2024, representing approximately 5.8 years of natural outdoor exposure. The logs were stacked in superimposed rows, with all test specimens sourced exclusively from the lowest row in direct contact with the ground. Throughout the exposure period, the bark remained intact, and the logs were continuously subjected to natural weathering without artificial covers or chemical preservative treatments. To further characterise the thermal environment during storage, 2 m air-temperature data from the ERA5 reanalysis dataset were evaluated for the period between 28 January 2019 and 2 December 2024. A freeze–thaw day was defined as a calendar day with a daily minimum air temperature ≤ 0 °C and a daily maximum air temperature > 0 °C, identifying 470 freeze–thaw days during exposure [34]. This metric served as an indicator of environmental freeze–thaw exposure rather than a direct measurement within the wood structure.

The second group (Control), established to provide baseline reference data, consisted of freshly harvested, healthy, and defect-free trees collected from the same location under equivalent growing conditions as the stored materials. The control logs were harvested in December 2024 and processed immediately without any storage period to prepare specimens for all subsequent analyses.

Log conversion and specimen preparation were performed at a local sawmill in accordance with TS ISO 3129:2019 [35]. A single tree stem (log) was utilised for each species–condition combination, yielding four primary sampling units: one control and one stored log for European hornbeam, and one control and one stored log for Oriental beech. Only sapwood material was selected. To minimise variations associated with maturity and tree dimensions, all selected logs fell within a narrow diameter range of 35–38 cm. Because the control and stored materials originated from different trees, they were not paired at the tree level. All test specimens for FTIR, XRD, TGA, SEM, and mechanical evaluations were prepared from their respective primary logs. Following preparation, all specimens were visually inspected, retaining only those free from obvious macroscopic defects, such as visible decay, severe cracking, and insect galleries. During this selection process, four European hornbeam specimens prepared for compression testing were excluded due to visible cracking and replaced with sound specimens from the same stored log. This selection procedure was applied consistently across all analytical and mechanical tests to ensure that the measured responses reflected intrinsic material changes. This approach enabled the assessment of the residual structural integrity and material properties of wood that remained suitable for service after long-term outdoor storage, minimising the influence of isolated defects to focus on residual performance rather than the maximum severity of visible deterioration.

Prior to physical and mechanical testing, all specimens were conditioned in a climate chamber at 20 ± 2 °C and 65 ± 5% relative humidity until an equilibrium moisture content of approximately 12% was achieved.

### 2.2. Attenuated Total Reflectance–Fourier Transform Infrared Spectroscopy (ATR-FTIR) Analysis

ATR-FTIR analysis was conducted to characterise relative changes in the chemical constituents of the wood samples, particularly cellulose, hemicellulose, and lignin-related functional groups. For each experimental group, a homogenised wood-flour composite sample was prepared from the respective primary log and analysed using an attenuated total reflectance (ATR) accessory coupled to a Shimadzu IRTracer-100 FTIR spectrometer (Shimadzu Corporation, Kyoto, Japan). Accordingly, four representative composite samples were analysed in total, corresponding to the control and stored conditions of each species. Spectra were recorded in absorbance mode over the range of 4000–400 cm^−1^ at a spectral resolution of 4 cm^−1^ using 32 scans per spectrum. Background spectra were collected prior to each measurement and automatically subtracted from the sample spectra. Baseline correction and spectral smoothing were subsequently applied to improve spectral quality.

The characteristic absorption bands were evaluated using relative band-intensity ratios $\left(I_{band}/I_{1502}\right)$. The band at 1502 cm^−1^, assigned to the aromatic skeletal vibrations of lignin, was selected as the internal spectral reference because it is a relatively stable lignin-associated band and is commonly used for the relative normalisation of wood FTIR spectra [36,37]. This approach allowed changes in the absorption bands to be evaluated on a common relative basis rather than from absolute absorbance values.

Consequently, the calculated band-intensity ratios were interpreted as relative spectral responses rather than absolute concentration measurements. As a single representative composite sample was analysed for each experimental group, the FTIR results were evaluated as a comparative semi-quantitative spectroscopic assessment, and no inferential statistical analysis was performed. Minor absorption bands observed within the 2300–1800 cm^−1^ region, originating from the ATR crystal, were excluded from spectral interpretation.

### 2.3. X-Ray Diffraction (XRD) Analysis

X-ray diffraction (XRD) analysis was performed to determine the crystalline arrangement of cellulose in the wood samples and to calculate the crystallinity index (CrI). Wood particles, approximately 0.5 mm in size, were utilised for the analyses. To eliminate the effects of free water on peak broadening and background noise, the samples were oven-dried at 103 ± 2 °C to constant mass and stored in a desiccator until measurement.

XRD measurements were carried out using a Rigaku SmartLab diffractometer (Rigaku Corporation, Tokyo, Japan). CuKα radiation (λ = 1.5418 Å) was employed as the X-ray source, operating at 40 kV and 30 mA. A nickel (*Ni*) filter was used to suppress CuKβ radiation, and no additional monochromator was applied. Diffraction patterns were recorded in *θ*/2*θ* scanning mode over a 2*θ* range of 5–80°, with a step size of 0.040° and a scanning speed of 3°·min^−1^. A single representative diffraction pattern was obtained for each experimental group; therefore, the XRD data were evaluated as a comparative assessment of crystallographic trends.

The CrI was calculated from X-ray diffraction patterns using the Segal empirical method, which is widely applied to provide a comparative, semi-quantitative assessment of cellulose crystallinity rather than an absolute crystallographic quantification [38]. This approach is based on the maximum diffraction intensity of the (002) plane of cellulose I (*I*_002_) and the minimum intensity corresponding to the amorphous region (*I_am_*). The calculation was performed using Equation (1):

(1) $$CrI(\%)=\frac{I_{002}-I_{am}}{I_{002}}\;\times \;100$$

Here, *I*_002_ represents the maximum diffraction intensity of the (002) crystalline plane of cellulose I observed at approximately 2*θ* = 22.5°, while *I_am_* denotes the minimum intensity associated with the amorphous region at approximately 2*θ* = 18.0°.

### 2.4. Thermal Analysis (TGA/DTG)

The thermal decomposition behaviour and thermal stability of the wood samples were determined using thermogravimetric analysis (TGA) and derivative thermogravimetry (DTG) with a Hitachi STA-7300 instrument (Hitachi High-Tech Science Corp., Tokyo, Japan). Prior to analysis, wood materials from each group were ground using a laboratory mill. The resulting wood flours were homogenised by mechanical mixing to obtain representative and uniform fractions.

Approximately 2.5 mg of sample was weighed for each measurement. High-purity alumina (*Al*_2_*O*_3_) crucibles were employed as sample holders, with an empty crucible used as a reference. The analyses were conducted under a nitrogen (*N*_2_) atmosphere at a flow rate of 100 mL·min^−1^ to prevent oxidative reactions and to monitor pyrolytic degradation behaviour. The samples were heated from 25 °C to 810 °C at a constant heating rate of 20 °C·min^−1^.

Mass-loss profiles and residual mass were determined from the TGA curves, whereas the DTG curves were used to identify the principal degradation stages of hemicellulose, cellulose, and lignin, together with their corresponding maximum degradation temperatures (*T_max_*). With a single representative TGA/DTG run performed for each experimental group, the thermal data were evaluated as a comparative assessment of overall pyrolytic behaviour.

### 2.5. Scanning Electron Microscopy (SEM) Analysis

Scanning electron microscopy (SEM) (TESCAN, Brno, Czech Republic) was performed to investigate the microstructural characteristics of wood after long-term outdoor storage, with particular emphasis on cell-wall deformation, microcracking, lamellar separation, and other morphological features associated with environmental exposure. The observations obtained from the stored samples were interpreted with reference to the characteristic anatomical features of beech and hornbeam wood reported in the literature.

For each stored species, representative wood blocks (10 × 10 × 10 mm) were prepared from the outermost sapwood layers immediately beneath the bark on freshly exposed cross-sectional surfaces, ensuring the inclusion of the primary degradation zone. To facilitate sectioning, the samples were boiled in distilled water until saturation. Subsequently, they were immersed in absolute ethanol for 20 days to remove water and to stabilise the structure [39]. After dehydration, transverse, radial, and tangential sections with a thickness of 15–20 µm were obtained using a Reichert sliding microtome.

The prepared sections were coated with a 10 nm conductive layer of gold (Au)–palladium (Pd) alloy (60% Au, 40% Pd) using a vacuum sputter coater to enhance electrical conductivity and prevent charging artefacts. SEM imaging was carried out using a TESCAN MAIA3-XMU instrument (TESCAN, Brno, Czech Republic) at an accelerating voltage of 10 kV, employing a secondary electron (SE) detector sensitive to surface topography. Multiple observation fields were examined for each anatomical section, and representative micrographs illustrating recurrent microstructural features were selected to ensure comparability.

### 2.6. Compressive Strength Parallel to Grain Tests

Mechanical performance of the wood samples was evaluated through compressive strength parallel to grain and modulus of elasticity (MOE) tests. A total of 80 specimens were used, with 20 test specimens for each experimental group. The specimens were prepared with dimensions of 20 × 20 × 30 mm (longitudinal direction: 30 mm) and conditioned at 20 ± 2 °C and 65 ± 5% relative humidity until reaching an equilibrium moisture content of approximately 12%.

Mechanical tests were conducted in accordance with TS ISO 13061-17:2017 using a 10-ton universal testing machine (UTEST Material Testing Equipment, Ankara, Türkiye) [40]. The crosshead speed was set to 2 mm·min^−1^ to ensure failure occurred within 90 ± 30 s. Compressive strength parallel to grain (*σ*_β_) was calculated using the maximum load at failure (*F_max_*) according to Equation (2):

(2) $$\sigma_{\beta }=\frac{F_{max}}{a\cdot b}\;$$

where *σ*_β_ is the compressive strength parallel to grain (MPa), *F_max_* is the maximum load at failure (N), and *a* and *b* represent the width and thickness of the specimen cross-section (mm), respectively.

The modulus of elasticity (MOE) was determined from the linear region of the compressive stress–strain curve. Compressive stress was calculated by dividing the applied load by the initial cross-sectional area, whereas strain was calculated by dividing the recorded axial deformation by the initial specimen length (30 mm), which was used as the gauge length. Within this linear region, the maximum stress-to-strain ratio ($\sigma_{i}/\varepsilon_{i}$) for each specimen was taken as the representative MOE value. The mean MOE for each experimental group was calculated as the arithmetic average of the individual specimen values. No separate compliance correction was applied. MOE was calculated using Equation (3):

(3) $$MOE=\frac{\sigma_{i}}{\varepsilon_{i}},\;i\in L$$

where MOE is the modulus of elasticity (MPa), *σ_i_* is the compressive stress (MPa), *ε_i_* is the corresponding compressive strain (mm mm^−1^), and *L* denotes the approximately linear region of the stress–strain curve.

Statistical analyses were performed to evaluate compressive strength and MOE data. Normality was assessed using the Shapiro–Wilk test, while homogeneity of variances was verified using Levene’s test. A two-way analysis of variance (two-way ANOVA) was applied, considering wood species (European hornbeam and Oriental beech) and storage condition (control and stored) as fixed factors. Main effects and interaction effects were evaluated at a 95% confidence level, with statistical significance set at *p* < 0.05. Coefficients of variation (CV, %) were also calculated to assess data homogeneity. Post hoc comparisons were performed using Tukey’s HSD test when significant differences were detected.

## 3. Results and Discussion

### 3.1. ATR-FTIR Spectroscopic Analysis and Functional Group Characterization

**Figure 1:**
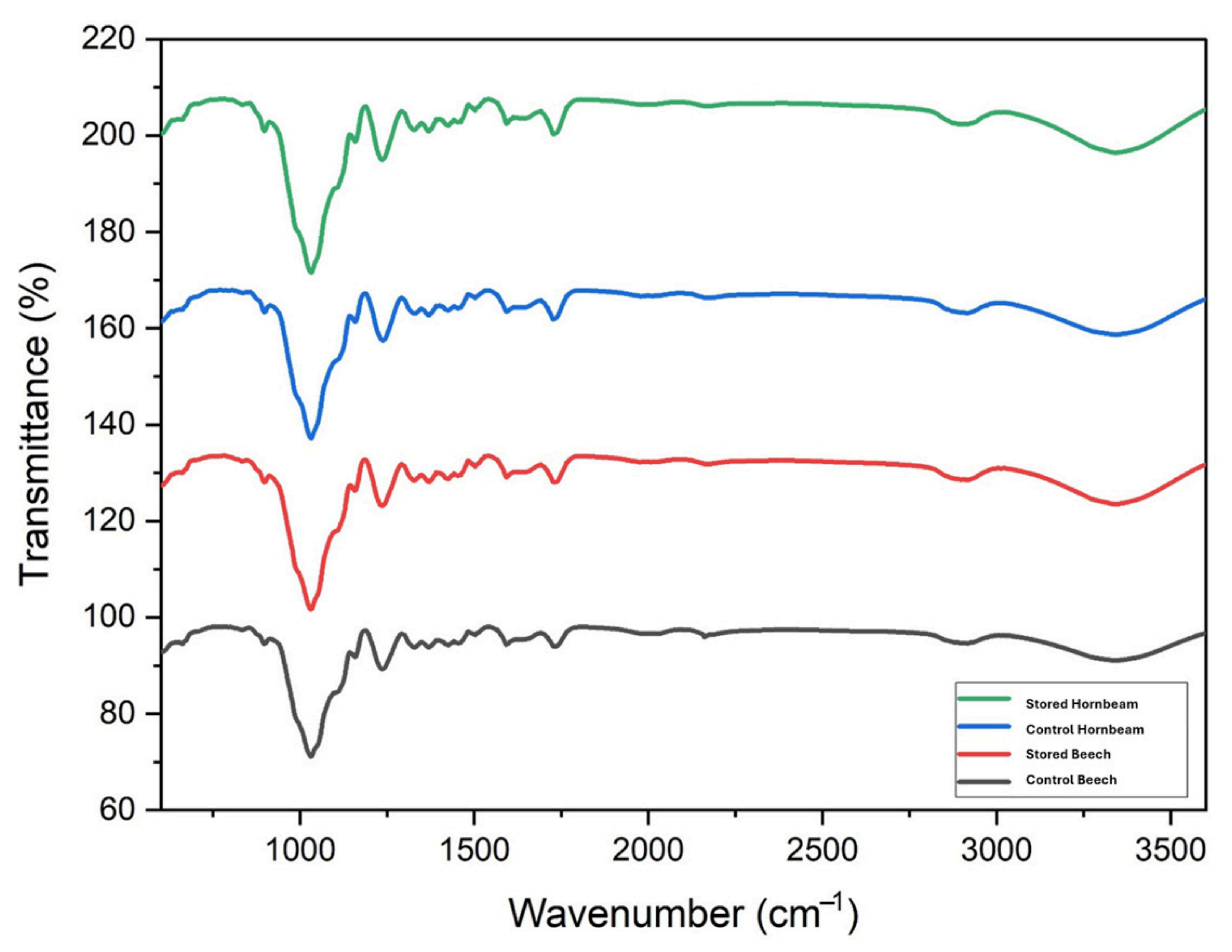
ATR-FTIR spectra of control and stored European hornbeam and Oriental beech wood samples.

ATR-FTIR spectra of control and naturally stored European hornbeam and Oriental beech wood samples are presented in Figure 1 in transmittance mode for visual comparison of the characteristic absorption band profiles. To enable quantitative evaluation of relative chemical changes in functional groups, the spectral data were converted to absorbance values.

The absolute absorbance intensities of the characteristic bands, together with the relative band intensity ratios normalised to the 1502 cm^−1^ band associated with aromatic skeletal vibrations of lignin ($I_{band}/I_{1502}$), are presented in Table 2.

A visual comparison of the transmittance spectra in Figure 1 showed that the overall spectral profiles of both species remained largely unchanged after long-term storage, with no appearance of new characteristic bands or complete disappearance of existing bands. However, the normalised data presented in Table 2 indicate that long-term outdoor exposure induced varying degrees of chemical alterations and structural rearrangements in the cell wall polymers. Since the 1502 cm^−1^ band, associated with lignin aromatic skeletal vibrations, is known to remain relatively stable during natural ageing processes [36,37], the extent of chemical degradation was evaluated based on the relative absorbance ratios normalised to this band.

The broad absorption band observed at approximately 3340 cm^−1^ is attributed to O–H stretching vibrations originating from hydroxyl groups in cellulose, hemicellulose, and lignin. When evaluated based on lignin-normalised spectra, the relative intensity of the O–H band decreased from 3.03 to 2.76 in hornbeam wood, whereas a slight increase from 2.48 to 2.66 was observed in beech wood. The reduction observed in hornbeam may be associated with the reorganisation of hydrogen bonding networks during prolonged environmental exposure, involvement of hydroxyl groups in oxidative reactions, and structural modifications in the carbohydrate matrix. In contrast, the slight increase in beech suggests an enhanced accessibility of hydroxyl groups as a result of structural rearrangements within the cell wall polymers. Similar trends have also been reported for aged and heat-treated wood materials [41,42].

The weak band observed at approximately 2916 cm^−1^ corresponds to C–H stretching vibrations of methyl and methylene groups. The normalised spectral data indicate a slight increase in band intensity from 1.47 to 1.56 in beech wood, whereas a decrease from 1.81 to 1.53 was observed in hornbeam. However, the limited magnitude of these changes suggests that long-term outdoor storage had no pronounced effect on the overall integrity of aliphatic structures in the wood cell wall.

The band observed at 1726 cm^−1^, associated with acetyl and ester carbonyl groups of xylan chains in hemicellulose, is considered a key indicator for assessing the degree of biotic and abiotic degradation in wood polymers [43]. In Figure 1, this band appears as a weak shoulder in the transmittance spectra. The normalised spectral data reveal a pronounced decrease in the $I_{1726}/I_{1502}$ ratio from 2.17 to 1.95 in stored hornbeam wood, whereas beech wood exhibited a nearly stable value (1.65 to 1.67). The reduction observed in hornbeam has been widely associated with deacetylation reactions, partial hydrolysis, and hydrolytic degradation processes occurring primarily in hemicellulose fractions [43,44], while the relatively stable behaviour in beech suggests a lower extent of chemical modification under identical storage conditions.

The aromatic skeletal vibrations of lignin, represented by the bands at 1592 cm^−1^ and 1502 cm^−1^, remained largely stable in both wood species, indicating the overall preservation of the lignin network during long-term storage. According to the normalised data, the $I_{1592}/I_{1502}$ ratio decreased slightly after storage, from 1.53 to 1.43 in beech and from 1.72 to 1.49 in hornbeam. The relatively small changes in the lignin-related absorption bands suggest that the chemical alterations associated with long-term outdoor storage were limited.

The spectral profiles revealed a dominant absorption band at 1031 cm^−1^, corresponding to C–O stretching vibrations in cellulose and hemicellulose, which is typically associated with the carbohydrate-rich matrix and exhibits the highest absorption (lowest transmittance) in this region. Normalised spectral data showed a decrease in the $I_{1031}/I_{1502}$ ratio from 8.99 to 8.27 in beech wood and from 9.88 to 8.97 in hornbeam wood. Consistently, a general reduction in the relative intensities of other carbohydrate-related bands (1427, 1370, 1327, 1234, and 1157 cm^−1^) was observed.

In contrast, the band at 897 cm^−1^, attributed to β-(1→4)-glycosidic linkages in amorphous cellulose, exhibited only minor changes following storage, with a slight increase in the normalised band intensity in both species. Compared with the more pronounced changes observed in hemicellulose-related bands, these results suggest that the cellulose backbone remained largely preserved during long-term outdoor exposure.

Overall, these findings indicate that the carbohydrate fraction of wood, particularly hemicellulose and amorphous cellulose regions, is more susceptible to environmental degradation than the lignin matrix. The results are consistent with previous studies reporting that polysaccharide components are more strongly affected by biotic and hydrolytic degradation during natural ageing processes [43].

### 3.2. X-Ray Diffraction (XRD) Analysis Results

**Figure 2:**
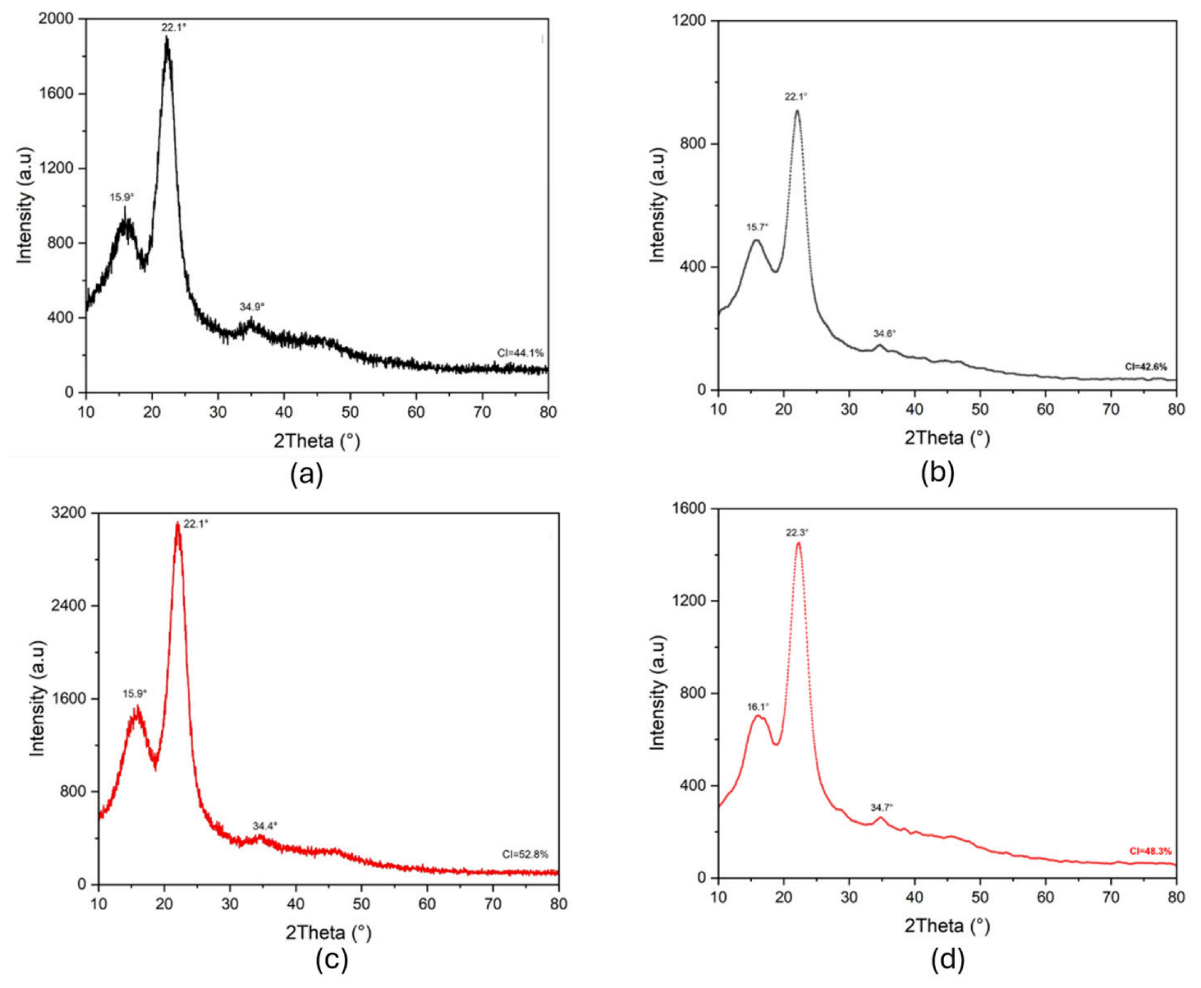
XRD patterns of hornbeam and beech wood samples: (**a**) control hornbeam; (**b**) stored hornbeam; (**c**) control beech; (**d**) stored beech.

X-ray diffraction (XRD) patterns of the control and stored hornbeam and beech wood samples revealed the characteristic reflections associated with the cellulose I crystalline structure (Figure 2). In all four representative patterns, three principal reflections were observed at approximately 2*θ* = 16°, 22°, and 34°.

The peak at ~16° corresponds to the overlapping $(1\bar{1}0)/\left(110\right)$ crystallographic planes, while the dominant reflection at ~22° is assigned to the (002) plane, representing the main crystalline orientation of cellulose I. The high-angle reflection at ~34° is attributed to the (040) plane. Among these, the (002) reflection exhibited the highest intensity in all samples, whereas the (040) peak showed comparatively lower intensity, consistent with the typical diffraction behaviour of lignocellulosic materials [45,46,47].

The crystallinity index (CrI) values represent relative changes in cellulose crystallinity rather than absolute crystallinity, depending on the applied evaluation method [47]. Variations in crystallite size may induce internal lattice strain, leading to minor shifts in diffraction peak positions [48]. In this context, small shifts in peak positions are associated with changes in the d-spacing of crystal planes according to Bragg’s law [49].

The diffraction pattern of the stored hornbeam sample retained the overall cellulose I diffraction profile of the control sample, although minor and heterogeneous variations in peak position and relative intensity were observed. The slight tendency of some reflections toward lower 2*θ* values is consistent with an increase in interplanar spacing and may indicate partial lattice relaxation within the crystalline domains. In the context of long-term outdoor exposure, this interpretation is compatible with the potential influence of repeated moisture adsorption–desorption cycles and associated residual physicochemical stresses in the cell-wall structure. However, because the observed changes were small and replicate-based measurement uncertainty was not independently determined, this interpretation is considered a plausible structural explanation rather than definitive crystallographic proof. The principal reflection near 22° remained comparatively stable, suggesting relative resistance of the dominant cellulose crystalline planes to the storage environment [50,51].

For beech wood, a slight shift in the diffraction peaks toward higher 2*θ* angles was observed relative to the control, which might indicate a slight reduction in interplanar spacing and a tentatively more compact cellulose crystallite arrangement than in hornbeam. This behaviour may be associated with irreversible removal of bound water from the cell wall during long-term outdoor exposure, as well as micro-compressive stresses induced by repeated wetting–drying cycles. Such cyclic moisture variations can promote localised densification of cellulose microfibrils, leading to minor contractions within the crystalline lattice.

As a result of long-term storage, a general decreasing trend in CrI values was observed in both species. Specifically, storage resulted in a minor relative reduction of 3.4% in the crystallinity index of hornbeam wood, whereas a more pronounced relative decrease of 8.5% was observed for beech wood. This reduction suggests that environmental effects are not limited to amorphous components of the cell wall but also extend to the surface and boundary regions of cellulose crystallites. Prolonged exposure to hydrolytic and oxidative processes, together with possible biological degradation, may lead to cleavage of glycosidic bonds, resulting in partial decrystallisation of cellulose structures. In addition, repeated moisture and temperature fluctuations may contribute to fatigue-induced disruption of microfibrillar order, thereby further reducing overall crystallinity [50,51,52].

The more limited reduction in CrI observed in hornbeam wood suggests a higher structural stability of its crystalline cellulose domains under long-term environmental exposure. This microstructural stability is also supported by FTIR results, where carbohydrate-related spectral features in hornbeam exhibited better preservation of overall band profiles. Such behaviour may be attributed to anatomical characteristics, including higher fibre density and thicker cell walls, which can act as a protective barrier against external degradation processes [22,53,54].

In contrast, the more pronounced CrI reduction observed in beech wood may be associated with its relatively more porous anatomical structure and wider lumen dimensions [55], which can facilitate the penetration of acidic agents and fungal enzymes into the cell wall matrix. Similar trends in crystallinity changes induced by ageing, thermal modification, and environmental exposure in hornbeam and beech woods have also been documented in related research [2,5,51,56,57,58,59].

### 3.3. Thermal Analysis (TGA/DTG) Results

**Figure 3:**
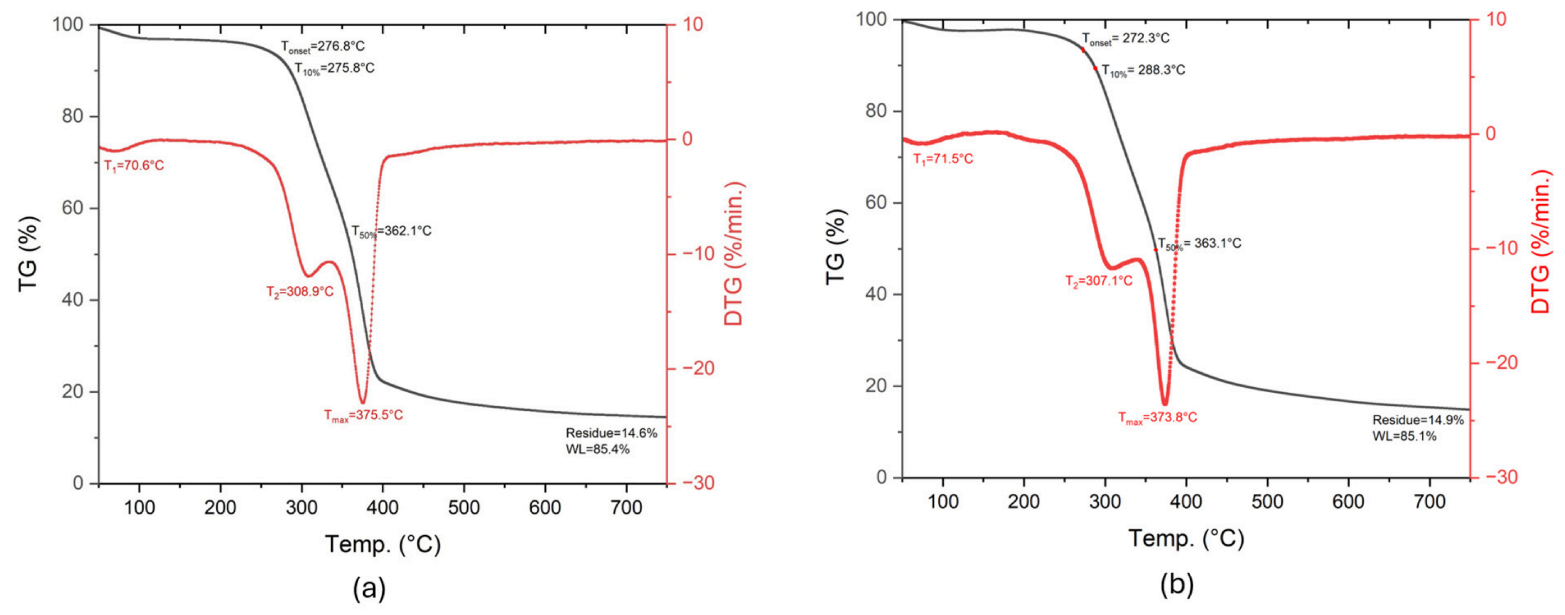
TGA/DTG curves of hornbeam wood samples: (**a**) control sample; (**b**) stored sample.

The TGA and DTG curves of control and stored hornbeam wood samples (Figure 3) exhibited broadly similar pyrolytic behaviour; however, measurable differences were observed in several characteristic thermal parameters. In both samples, the initial mass loss (*T*_1_) occurred at approximately 70 °C and was associated with the removal of bound water and low-molecular-weight volatile compounds [47,60,61]. The comparable mass loss in this stage indicates similar initial moisture content and loosely bound volatile fractions in both samples.

The main pyrolysis region, attributed to the overlapping degradation of hemicellulose, cellulose, and partially degraded lignin, was observed in the temperature range of approximately 300–380 °C [2,62]. The onset temperature (*T_onset_*) decreased slightly from 276.8 °C in the control sample to 272.3 °C in the stored sample, indicating a marginal reduction in thermal stability. This behaviour is consistent with FTIR observations, particularly the variations in hemicellulose-related bands, and may be related to the preferential hydrolysis and degradation of amorphous carbohydrate fractions, which are more thermally labile [63,64,65].

In contrast, the temperature corresponding to 10% mass loss (*T*_10_%) increased from 275.8 °C in the control sample to 288.3 °C in the stored sample. This shift may be attributed to the partial depletion of thermally labile constituents during long-term storage, resulting in a residual structure with comparatively higher thermal resistance in this temperature region [66].

In the DTG curves, the secondary shoulder peak associated with hemicellulose decomposition (*T*_2_) in the stored hornbeam sample occurred at only 1.8 °C lower temperature compared to the control sample. This marginal shift indicates that the polymeric structure of hornbeam wood largely retained its thermal stability under long-term outdoor exposure. Nevertheless, the observed difference suggests that limited hydrolytic chain scission and molecular disordering may have occurred in the hemicellulose fraction, which is considered the least ordered and most environmentally sensitive component of the cell wall [29,67,68].

The maximum decomposition rate temperature (*T_max_*) was recorded at 375.5 °C for the control sample and 373.8 °C for the stored sample, indicating only a minor reduction. In addition, a slight broadening of the main DTG peak was observed in the stored specimen. This behaviour suggests that thermal degradation reactions occurred over a somewhat wider and more heterogeneous temperature range, likely due to limited structural disorder and chemical modifications in amorphous carbohydrate fractions during storage [64].

The residual char yield was 14.9% for the stored sample and 14.6% for the control sample, indicating close agreement between the two profiles. Accordingly, the char data do not provide evidence of a substantial change in the overall residual mass-forming behaviour of hornbeam under the investigated conditions. The values were also comparable to previously reported char yields of approximately 14.8% for sound hornbeam wood under similar conditions [29]. The observed thermal variations are therefore more likely associated with localised modifications in amorphous and surface regions rather than bulk structural transformations [65].

**Figure 4:**
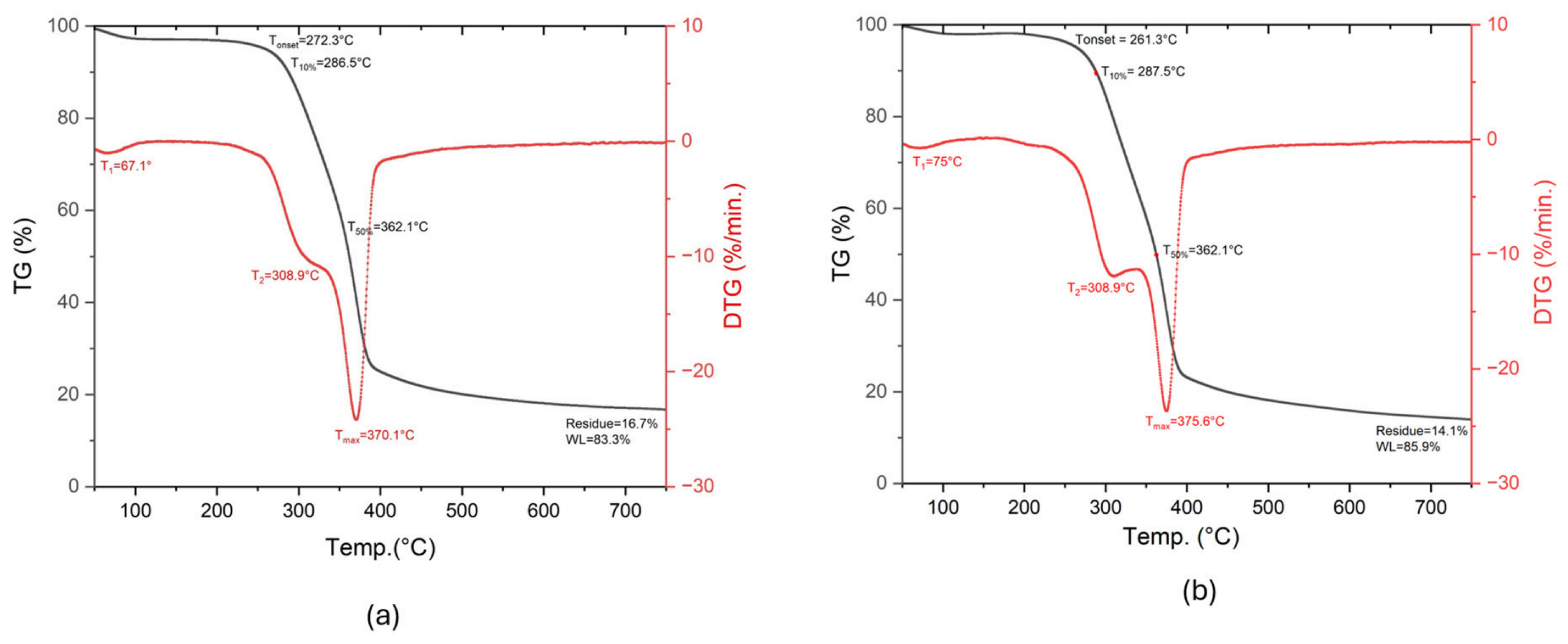
TGA/DTG curves of beech wood samples: (**a**) control sample; (**b**) stored sample.

The TGA and DTG results of beech wood (Figure 4) indicated a more pronounced effect of long-term storage on thermal stability and decomposition behaviour compared to hornbeam wood. In both samples, the initial mass loss region (*T*_1_) occurred within approximately 65–75 °C; however, this region in the stored sample showed a slight shift toward higher temperatures. This behaviour may be associated with limited densification in the crystalline domains identified in the XRD analysis, as well as possible structural rearrangements within the cell wall matrix, which could influence the kinetics of bound water release. In addition, chemical modifications in carbohydrate components during storage may alter the interaction between hydroxyl groups and water molecules, thereby affecting moisture desorption behaviour [69,70,71].

The main pyrolysis region occurred in the range of approximately 300–380 °C for both samples. The onset temperature (*T_onset_*) decreased from 272.3 °C in the control sample to 261.3 °C in the stored sample, indicating a notable reduction in thermal stability. This decrease is consistent with FTIR observations, particularly changes in the 1726 cm^−1^ band, and may be attributed to deacetylation and partial hydrolytic degradation of hemicellulose fractions [63,65]. The hydrolysis of acetyl groups and the subsequent release of acetic acid may promote autocatalytic cleavage reactions within polymer chains, thereby accelerating thermal decomposition at lower temperatures [17,67,72]. Despite these changes, the relatively stable *T*_10_% values suggest that the primary degradation behaviour of the cellulose matrix remains largely preserved.

The hemicellulose-related shoulder peak (*T*_2_) remained nearly unchanged at approximately 308.9 °C in both samples. In contrast, the maximum decomposition rate temperature (*T_max_*) increased by 5.5 °C in the stored sample, reaching 375.6 °C. The 8.5% reduction in crystallinity index observed in XRD analysis suggests partial degradation of thermally labile amorphous carbohydrate fractions during storage. This structural alteration may have led to a more pronounced relative contribution of lignin within the remaining matrix, which is thermally more resistant, potentially resulting in a more concentrated and sharper DTG peak profile [65,66].

The char yield decreased from 16.7% in the control sample to 14.1% in the stored sample. In comparison, a higher char yield of 19.6% has been reported for sound beech wood in the literature [72]. This reduction may indicate that storage-induced modifications are not limited to carbohydrate components but may also involve partial oxidative or hydrolytic alterations in lignin structures. Such modifications in lignin can influence pyrolysis pathways by promoting increased volatile formation, thereby reducing char yield during thermal degradation [65,73,74].

### 3.4. Scanning Electron Microscopy (SEM) Results

Following six years of outdoor storage, both hornbeam and beech wood generally retained their anatomical framework, with annual ring structure remaining regular and the samples preserving sufficient integrity during specimen preparation and microtome sectioning. No fragmentation or evidence of catastrophic structural collapse was observed. However, SEM examination revealed species-dependent localised microstructural alterations, indicating that prolonged storage affected cell-wall morphology without destroying the overall tissue architecture.

**Figure 5:**
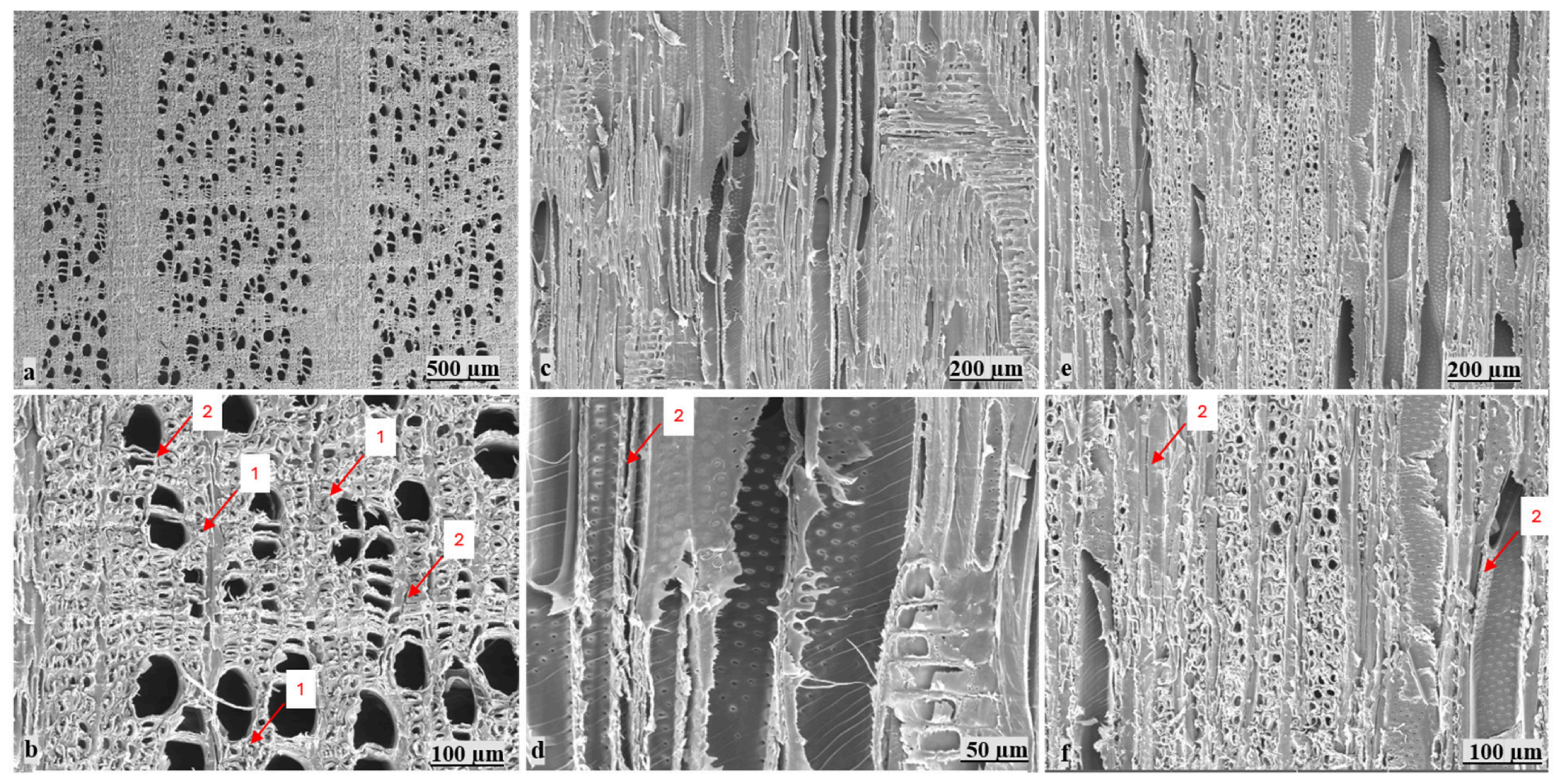
Representative scanning electron microscopy (SEM) micrographs of stored European hornbeam wood showing the transverse (**a**,**b**), radial (**c**,**d**), and tangential (**e**,**f**) anatomical sections. The numbered annotations indicate the principal microstructural features identified in the images: (1) microcracks, (2) cell-wall disruption.

Representative SEM micrographs of hornbeam wood are presented in Figure 5.

In all three anatomical planes, the hornbeam samples maintained a largely intact cellular organisation, with deterioration features remaining limited and spatially localised. In transverse sections (Figure 5a,b), fibre-wall continuity was generally preserved, although local surface separations, microcracks, flake-like detachments, and occasional lumen narrowing were detected. Slight geometric irregularities were also noted in some vessel elements.

In radial sections (Figure 5c,d), the longitudinal arrangement of the cells remained clearly recognisable, including the simple perforation plates of the vessel elements. Most pits retained their morphology and distribution, and the vessel–fibre interface showed no marked instability. Ray tissues remained well defined, while most fibres preserved visible lumens and largely continuous cell walls. Only occasional fine longitudinal separations parallel to the wall surface were observed.

Tangential sections of hornbeam (Figure 5e,f) likewise showed preserved ray organisation, with rays remaining visible as distinct vertical bands and without marked deformation. Ray boundaries were sufficiently intact to allow reliable morphological assessment. The absence of pronounced collapse, severe delamination, or extensive erosion supports the interpretation that the changes in hornbeam were mainly associated with mild physical and chemical weathering during environmental exposure.

**Figure 6:**
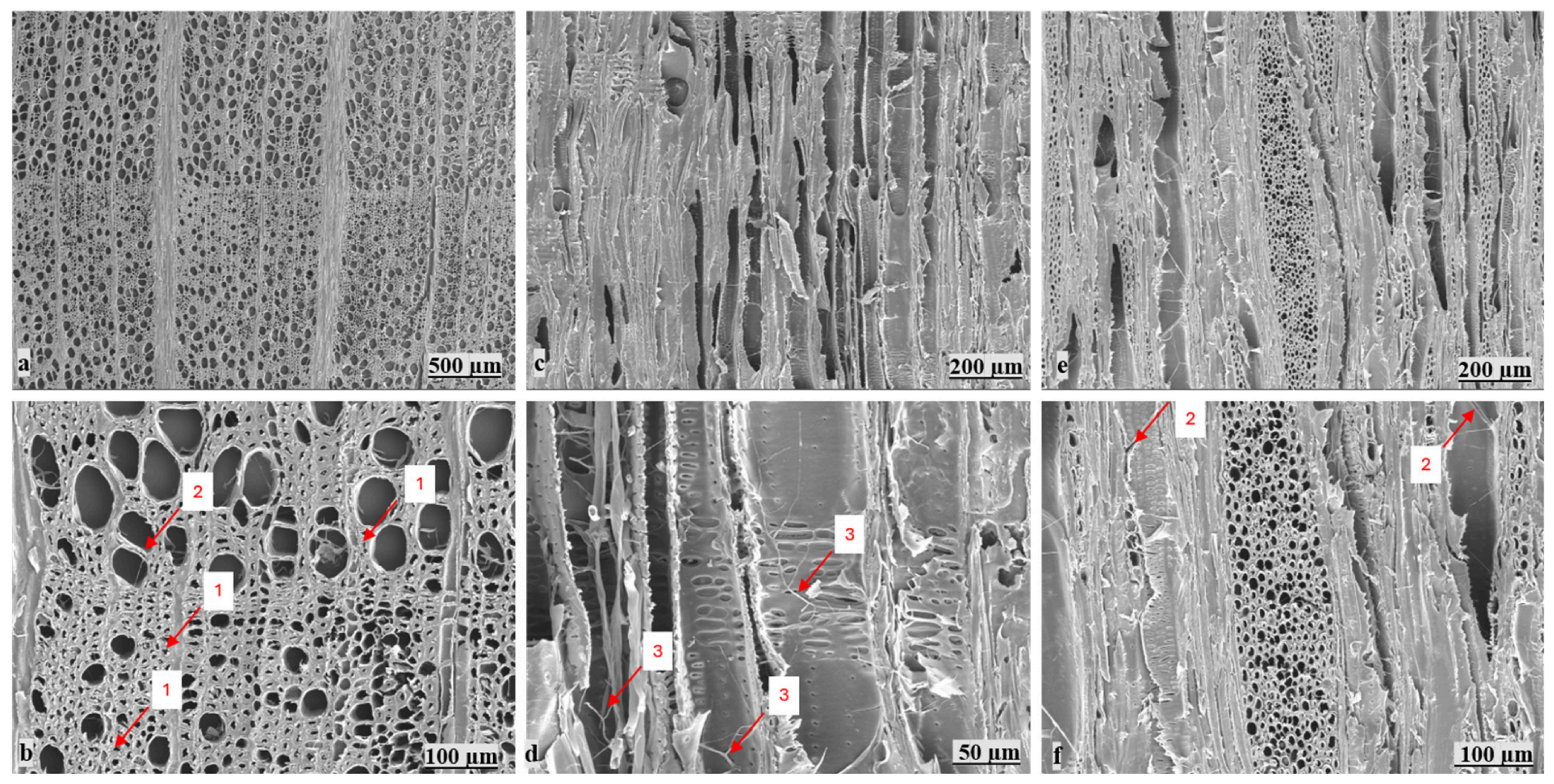
Representative scanning electron microscopy (SEM) micrographs of stored Oriental beech wood showing the transverse (**a**,**b**), radial (**c**,**d**), and tangential (**e**,**f**) anatomical sections. The numbered annotations indicate the principal microstructural features identified in the images: (1) microcracks, (2) cell-wall disruption, and (3) filamentous structures.

In contrast, the stored beech samples exhibited a broader and more heterogeneous pattern of alteration (Figure 6). Although the overall anatomical arrangement remained identifiable, the observed features pointed to greater local instability of the cell wall and lumen surfaces.

In transverse sections (Figure 6a,b), localised surface irregularities and occasional filamentous structures were present within certain cell lumens. While these features do not indicate severe collapse, they are consistent with limited biological and/or environmental activity.

The radial sections of beech (Figure 6c,d) showed the clearest evidence of deterioration. Partial cell-wall delamination, microcracks, exfoliation-like features, and local intercellular separation were observed, indicating weakening of inter-fibre cohesion. Vessel elements largely retained their general morphology, but local thinning around perforation edges and irregular erosion near pit regions were evident. Ray parenchyma cells displayed partial luminal collapse and local disruption of linear continuity. In some lumens, filamentous material, fibrillar fragments, and detached cellulosic debris were also observed.

Tangential sections of beech (Figure 6e,f) supported this interpretation. Ray morphology remained broadly recognisable, but minor separations between ray cells, localised wall disruptions in fibre-rich regions, and accumulated debris were evident. Filamentous structures were again detected within lumens, often concentrated around pit regions and extending transversely across the tissue. Their repeated association with pit-adjacent irregularities suggests that these microdomains may have been preferentially affected during storage-related deterioration.

Previous studies have shown that biological weathering, particularly by white-rot fungi, can promote delignification of the middle lamella and subsequent cell separation. The filamentous structures and cell-wall separations observed here are morphologically consistent with such deterioration patterns, especially in beech. However, SEM morphology alone does not provide definitive evidence of fungal colonization, and these features should therefore be interpreted cautiously as suggestive rather than conclusive indicators of biotic degradation [75,76,77,78,79].

These observations are consistent with the complementary chemical, crystalline, thermal, and mechanical findings obtained in the present study, collectively indicating that prolonged outdoor storage progressively modifies wood structure across multiple hierarchical levels.

### 3.5. Parallel-to-Grain Compressive Strength and Modulus of Elasticity Results

**Figure 7:**
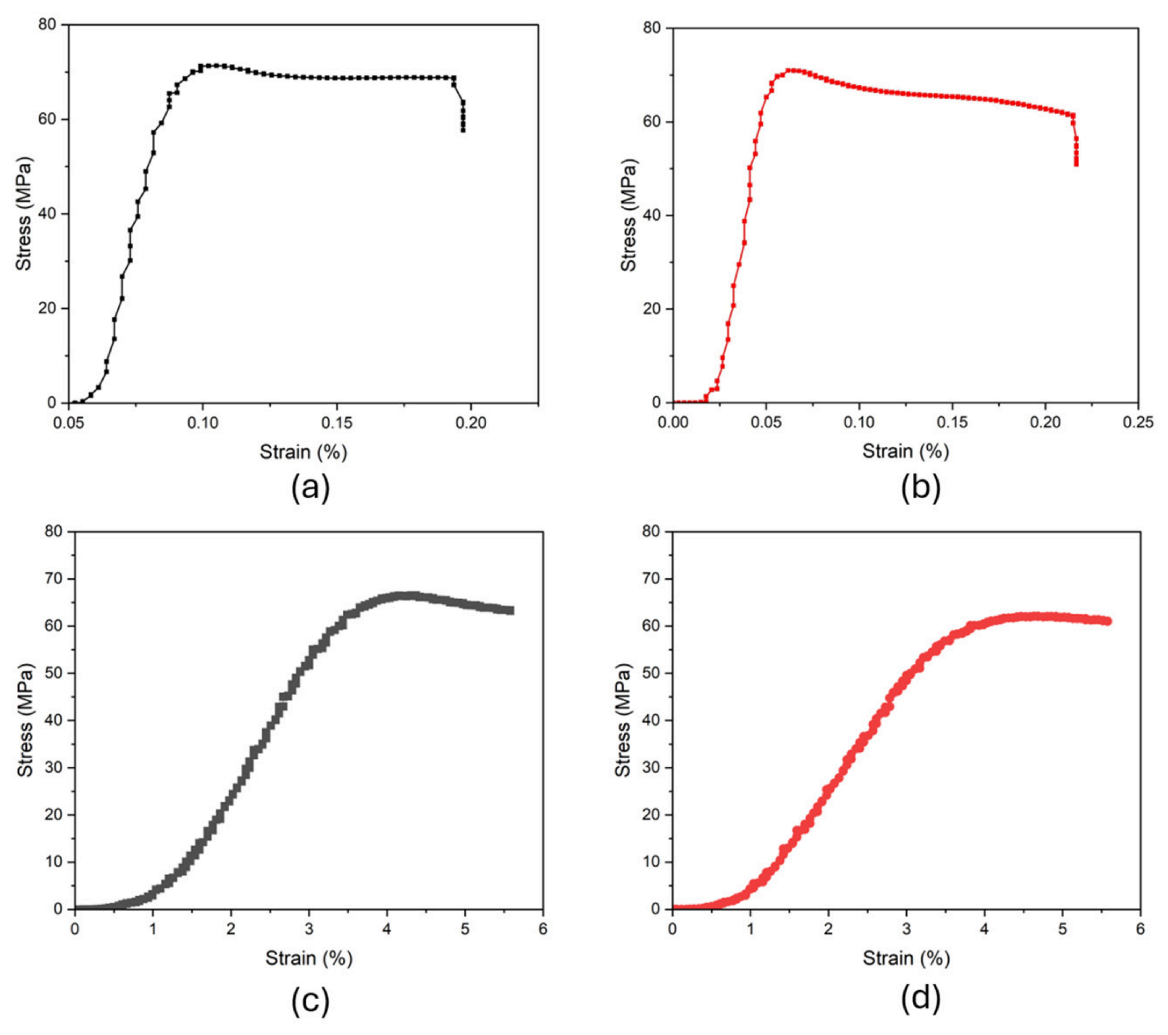
Stress–strain curves obtained under compression parallel to grain for control and stored hornbeam and beech wood specimens: (**a**) control hornbeam; (**b**) control beech; (**c**) stored hornbeam; (**d**) stored beech.

The compression strength parallel to the grain, modulus of elasticity, and stress–strain responses of control and long-term stored European hornbeam and Oriental beech specimens exhibited characteristic deformation and failure behaviours for each wood species. Representative stress–strain curves for each experimental group are presented in Figure 7. The strain axes are shown according to the data acquisition settings of the testing system for each representative specimen.

Both control and outdoor-stored specimens exhibited an initial linear elastic region, indicating that the cell walls effectively carried the applied load without macroscopic damage during the early stage of compression. In the control specimens, this elastic region was characterised by a steeper slope and a more distinct yield point, whereas the stored specimens displayed a lower slope, reflecting a reduction in stiffness associated with hydrothermal ageing and structural changes in the cell-wall polymers. This behaviour indicates a reduced macroscopic load-bearing efficiency and energy dissipation capacity after long-term storage. In this transition region, hornbeam exhibited a more irregular curve profile with localised fluctuations in stress, suggesting heterogeneous activation of micro-scale failure mechanisms such as fibre collapse or localised wall buckling. In contrast, beech showed a more stable deformation response up to the peak stress, with a smoother elastic–plastic transition and without a clearly defined yield point. Furthermore, the representative curves of the stored specimens exhibited a limited post-peak stress softening behaviour.

**Figure 8:**
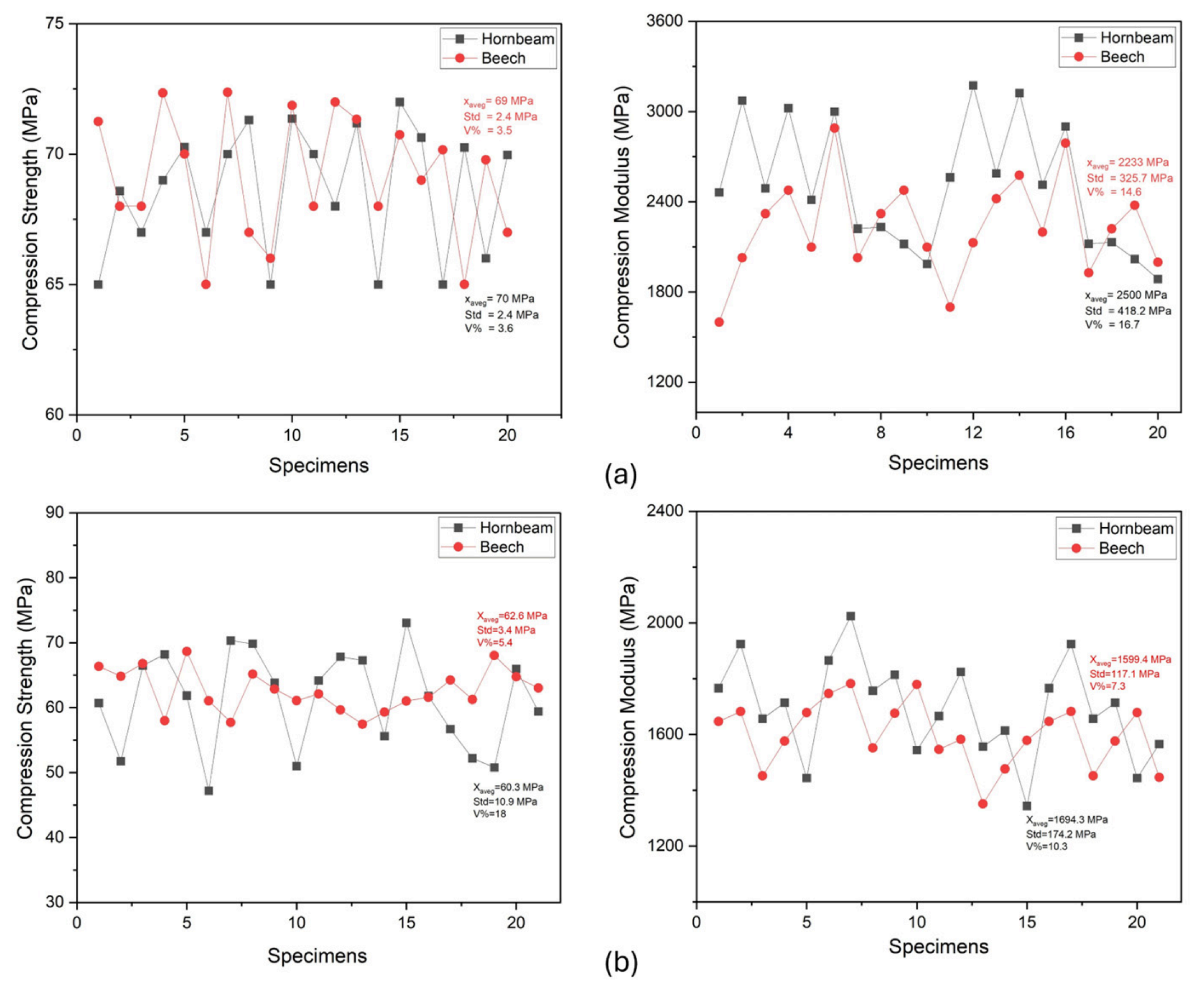
Distributions of compressive strength parallel to grain and modulus of elasticity of control and stored hornbeam and beech wood specimens: (**a**) control specimens; (**b**) stored specimens.

Figure 8 shows the distribution of specimen-based compressive strength and modulus of elasticity values, which, when evaluated together, clearly highlight the differences in mechanical strength and stiffness between the two wood species.

Test results indicated that the control specimens of hornbeam and beech exhibited average compressive strengths of 70.0 and 69.0 MPa, respectively. Both species also showed relatively low coefficients of variation (3.6% and 3.5%, respectively), indicating a uniform mechanical response among specimens. After approximately six years of outdoor storage, compressive strength decreased by 13.8% in hornbeam to 60.3 MPa, whereas beech exhibited a smaller reduction of 9.3%, reaching 62.6 MPa.

To support these observations, two-way analysis of variance (ANOVA) demonstrated that the storage period had a highly significant effect on both compressive strength and modulus of elasticity (*p* < 0.001). Tukey’s HSD post-hoc analysis confirmed significant reductions in both mechanical properties after storage within each species (*p* < 0.001). Furthermore, the stored hornbeam and beech specimens exhibited statistically comparable mechanical performance (*p* > 0.05), indicating that prolonged outdoor storage reduced the initial differences between the species and resulted in similar compressive strength and stiffness levels despite the overall deterioration of their mechanical properties (Table 3).

After storage, the coefficient of variation (*V*%) in compressive strength of hornbeam wood increased markedly from 3.6% in the control group to 18% in the stored group. Although the overall mean strength of hornbeam was partially retained, the material exhibited a highly unstable failure behaviour under load. Local structural defects, developed through repeated swelling–shrinkage cycles, led to premature failure of some specimens at relatively low stress levels, while others with fewer defects withstood considerably higher loads. This heterogeneity resulted in an increased standard deviation (±10.9 MPa).

In contrast, beech wood showed a narrow variability range in compressive strength in both control (*V* = 3.5%) and stored (*V* = 5.4%) conditions. This suggests that degradation in beech occurred more uniformly across the cell wall structure rather than through the formation of localised weak regions, resulting in consistently low variability in failure strength.

Modulus of elasticity results further support these trends. The control groups exhibited MOE values of 2500 MPa for hornbeam and 2233 MPa for beech, whereas after storage the values decreased by 32.2% to 1694.3 MPa in hornbeam and by 28.4% to 1599.4 MPa in beech wood. Although stored hornbeam maintained relatively higher MOE values on average compared to stored beech, indicating a more rigid initial response, the pronounced variability in this parameter revealed a low consistency in mechanical response among specimens under external loading. In contrast, stored beech wood, despite its lower absolute MOE values, exhibited a narrower distribution range, indicating a more stable and predictable stiffness behaviour.

According to relevant standardised studies, the compressive strength parallel to grain of sound European hornbeam wood has been reported as 72.3 MPa [21], while that of sound beech wood ranges between 67.4 and 73.3 MPa [27,59].

## 4. Conclusions

The findings of this study demonstrate that beech and hornbeam woods exposed to long-term open-air storage exhibit distinct degradation mechanisms and structural adaptation behaviours at both microscopic and macroscopic scales.

The integrated evaluation of TGA, XRD, SEM, FTIR, and mechanical test results indicates that hornbeam wood largely preserves its chemical and thermal constituents; however, it exhibits pronounced heterogeneity in mechanical behaviour due to micro-crack formation induced by repeated hygroscopic swelling–shrinkage cycles. FTIR analyses further confirmed this chemical degradation through evident changes in bands associated with carbohydrate fractions, while lignin-related structural signals remained comparatively stable. In contrast, although beech wood also exhibited thermo-oxidative degradation and partial reduction in crystalline order, the relative depletion of amorphous carbohydrate fractions led to a comparatively more dominant cellulose crystalline structure, resulting in a more homogeneous and predictable mechanical response.

These findings suggest that the response to storage conditions is governed not only by chemical composition changes but also by a multiscale mechanism controlled by anatomical structure and cell wall organization. In this context, hornbeam wood is primarily affected by physically induced damage (micro-crack formation and moisture-driven stress accumulation), whereas beech wood is governed more by biochemical degradation processes (interactions between hemicellulose and lignin and the crystalline/amorphous phase balance).

These differences highlight the importance of species-specific preservation strategies in storage management. For hornbeam logs, protective measures aimed at limiting physical environmental effects (moisture fluctuations and surface crack formation) should be prioritized, whereas for beech logs, strategies targeting biological and chemical degradation processes appear more critical.

Additionally, SEM and mechanical test data provide a directly usable dataset for micro-mechanical modeling of storage-induced performance losses. Similarly, TGA and XRD results can serve as quantitative indicators for evaluating the cascading use potential of logs that have reached the end of their service life or experienced quality degradation. Thermal decomposition parameters may be used to define processability limits in biocomposite manufacturing and to optimise thermochemical conversion processes such as pyrolysis and gasification, while changes in crystallinity may serve as indirect but meaningful predictors of enzymatic hydrolysis performance in biological degradation pathways.

Overall, open-air storage significantly limits the direct applicability of both species in load-bearing systems or high-performance engineering applications. Nevertheless, the findings clearly emphasize the importance of optimised time management in log storage yards and the necessity of species-specific protective strategies for maintaining material performance and improving biomass utilization efficiency.

## Figures and Tables

**Table 1:** Some physical and chemical properties of European hornbeam and Oriental beech wood.

Property	European Hornbeam	Oriental Beech	References
Density (ρ_12_, g·cm^−3^)	0.53–0.83	0.49–0.73	Hornbeam: [22,24,25] Beech: [26,27,28]
Cellulose (%)	47–48	50–54	Hornbeam: [29,30] Beech: [31,32]
Hemicelluloses (%)	32–35	19–21	Hornbeam: [29,30] Beech: [31,32]
Lignin (%)	18–20	20–24	Hornbeam: [29,30] Beech: [9,31]

**Table 2:** ATR-FTIR absorption bands, band assignments, absolute absorbance values, and normalised relative intensity ratios ($I_{band}/I_{1502}$) of control and stored wood samples.

Wavenumber (cm^−1^)	Band Assignment	Beech (Control) Abs/$I_{band}/I_{1502}$	Beech (Stored) Abs/$I_{band}/I_{1502}$	Hornbeam (Control) Abs/$I_{band}/I_{1502}$	Hornbeam (Stored) Abs/$I_{band}/I_{1502}$
3340	O–H stretching vibrations (cellulose, hemicellulose, lignin)	0.04071/2.48	0.05909/2.66	0.05343/3.03	0.06570/2.76
2916	C–H stretching vibrations (CH_2_ and CH_3_ groups)	0.02410/1.47	0.03474/1.56	0.03198/1.81	0.03655/1.53
1726	C=O stretching (acetyl and ester groups of hemicellulose/xylan)	0.02705/1.65	0.03704/1.67	0.03820/2.17	0.04656/1.95
1592	Aromatic skeletal vibrations of lignin (C=C)	0.02522/1.53	0.03172/1.43	0.03036/1.72	0.03560/1.49
1502	Aromatic skeletal vibrations of lignin (spectral reference band)	0.01644/1.00	0.02225/1.00	0.01762/1.00	0.02385/1.00
1427	C–H bending vibrations (cellulose/lignin)	0.02579/1.57	0.03383/1.52	0.02929/1.66	0.03802/1.59
1370	C–H bending vibrations in carbohydrates	0.02790/1.70	0.03664/1.65	0.03404/1.93	0.04365/1.83
1327	C–O stretching in crystalline cellulose	0.02794/1.70	0.03513/1.58	0.03218/1.83	0.04214/1.77
1234	C–O stretching of xylan (hemicellulose)	0.04908/2.99	0.06082/2.73	0.05884/3.34	0.07294/3.06
1157	C–O–C asymmetric stretching (cellulose/hemicellulose)	0.03722/2.26	0.04522/2.03	0.04024/2.28	0.05390/2.26
1031	C–O stretching (carbohydrate matrix: cellulose/hemicellulose)	0.14786/8.99	0.18402/8.27	0.17415/9.88	0.21385/8.97
897	β-(1→4)-glycosidic linkages in amorphous cellulose	0.02489/1.51	0.03695/1.66	0.03101/1.76	0.04332/1.82

**Table 3:** Compressive strength parallel to grain and modulus of elasticity values with statistical grouping.

Group	Compressive Strength (MPa)	MOE (MPa)
Hornbeam—Control	70.0 ± 2.4 ^a^	2500.0 ± 418.2 ^a^
Beech—Control	69.0 ± 2.4 ^a^	2233.0 ± 325.7 ^b^
Hornbeam—Stored	60.3 ± 10.9 ^b^	1694.3 ± 174.2 ^c^
Beech—Stored	62.6 ± 3.4 ^b^	1599.4 ± 117.1 ^c^

Different letters within columns indicate significant differences (Tukey’s HSD, *p* < 0.05).

## Data Availability

The data presented in this study are available upon request from the corresponding author.

## Abbreviations

The following abbreviations are used in this manuscript:

ATR-FTIR	Attenuated total reflectance Fourier-transform infrared spectroscopy
FTIR	Fourier-transform infrared spectroscopy
SEM	Scanning electron microscopy
XRD	X-ray Diffraction Analysis
CrI	Crystallinity index
TGA	Thermogravimetric analysis
DTG	Derivative thermogravimetry
MOE	The modulus of elasticity
MPa	Megapaskal
ρ_12_	Wood density at 12% moisture content
